# Mixed-metal nanoparticles: phase transitions and diffusion in Au–VO clusters†

**DOI:** 10.1039/d2fd00089j

**Published:** 2022-07-11

**Authors:** Wolfgang E. Ernst, Maximilian Lasserus, Daniel Knez, Ferdinand Hofer, Andreas W. Hauser

**Affiliations:** a Institute of Experimental Physics, Graz University of Technology Graz Austria wolfgang.ernst@tugraz.at andreas.hauser@tugraz.at; b Institute for Electron Microscopy and Nanoanalysis, Graz University of Technology Graz Austria

## Abstract

Nanoparticles with diameters in the range of a few nanometers, consisting of gold and vanadium oxide, are synthesized by sequential doping of cold helium droplets in a molecular beam apparatus and deposited on solid carbon substrates. After surface deposition, the samples are removed and various measurement techniques are applied to characterize the created particles: scanning transmission electron microscopy (STEM) at atomic resolution, temperature dependent STEM and TEM up to 650 °C, energy-dispersive X-ray spectroscopy (EDXS) and electron energy loss spectroscopy (EELS). In previous experiments we have shown that pure V_2_O_5_ nanoparticles can be generated by sublimation from the bulk and deposited without affecting their original stoichiometry. Interestingly, our follow-up attempts to create Au@V_2_O_5_ core@shell particles do not yield the expected encapsulated structure. Instead, Janus particles of Au and V_2_O_5_ with diameters between 10 and 20 nm are identified after deposition. At the interface of the Au and the V_2_O_5_ parts we observe an epitaxial-like growth of the vanadium oxide next to the Au structure. To test the temperature stability of these Janus-type particles, the samples are heated *in situ* during the STEM measurements from room temperature up to 650 °C, where a reduction from V_2_O_5_ to V_2_O_3_ is followed by a restructuring of the gold atoms to form a Wulff-shaped cluster layer. The temperature dependent dynamic interplay between gold and vanadium oxide in structures of only a few nanometer size is the central topic of this contribution to the Faraday Discussion.

## Abstract 2

Nanoparticles with diameters in the range of a few nanometers, consisting of gold and vanadium oxide, are synthesized by sequential doping of cold helium droplets in a molecular beam apparatus and deposited on solid carbon substrates.

## Graphical abstract

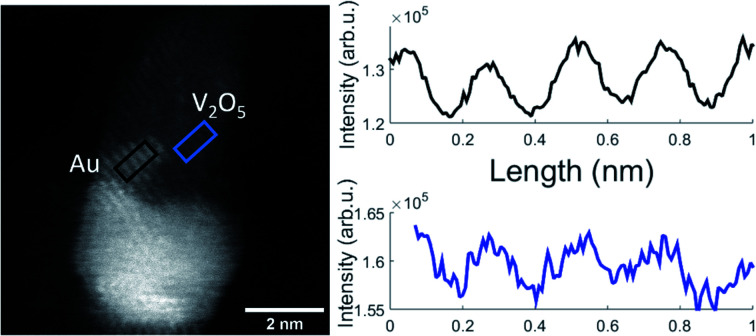

## 1 Introduction

With numerous potential applications, nanosized metal and metal oxide particles have become increasingly interesting also for fundamental research.^1^ Prospective applications range from the development of new catalysts and sensors^2–7^*via* battery electrode design^8^ to nano-optical and nano-electronic devices.^9,10^ Materials based on vanadium oxides have been particularly interesting for catalysis. Due to the different oxidation stages of vanadium, the chemical properties vary strongly, and nanostructured vanadium oxide, prepared in a specific oxidation state, is a highly desired but challenging goal.^11^ Battery electrodes could be improved by using two-dimensional V_2_O_5_ polymorphs as alkali intercalation compounds.^12,13^ Combinations with gold are considered promising for catalytic oxidation of hydrocarbons or CO^14,15^ as well as for enhanced sensing properties.^16^ Significant amounts of clusters can be synthesized from precursor molecules in solution by chemical methods yielding so-called ligand stabilized clusters, see for example ref. 17. These methods have the disadvantage that the influence of the ligand may alter the desired cluster properties. A solvent-free nanoparticle synthesis including some degree of structure and size control has been demonstrated by Palmer and co-workers.^18^ For the last decade, we have followed an approach to create clusters of atoms or molecules in a cold superfluid helium environment. The method is based on the development of an efficient source for generating superfluid helium droplets in a beam expansion. The droplets can serve as cold traps for the accumulation of other species to form clusters that may be deposited on solid substrates. As we have shown, nanoparticles of virtually any combination of elements can be produced in this way.^19^ Due to the inert environment of superfluid helium, they are free of any other chemicals or additives. With all processes including the deposition taking place in ultrahigh vacuum (UHV), contaminations of the produced samples are excluded. In this paper, we report the synthesis of mixed gold–vanadium oxide nanoparticles and their deposition on amorphous carbon substrates. The created samples were studied by electron microscopy, energy dispersive X-ray spectroscopy (EDXS), and electron energy loss spectroscopy (EELS). Our focus was set on the temperature dependence of the observed structures and corresponding phase transitions in the range from room temperature to about 650 °C. Prior to this work on the mixed nanoparticles, we have shown that clusters of vanadium pentoxide can be produced in superfluid helium droplets by accumulation of sublimated (V_2_O_5_)_2_ from a hot pick-up oven.^20^ V_2_O_5_ nanoparticles of sizes around 10 nm were clearly identified after deposition at room temperature by scanning transmission electron microscopy (STEM), EELS, and UV-visible absorption spectroscopy.^21^ This article is structured as follows. First, we provide the details of the helium droplet experiment and our measurement techniques. This section is then followed by a brief overview of our computational attempts to better understand the interactions between gold and vanadium oxide. In Section 4 we present the results of our temperature-dependent studies and discuss the observed structures and phase transitions.

## 2 Experimental details

**Fig. 1:**
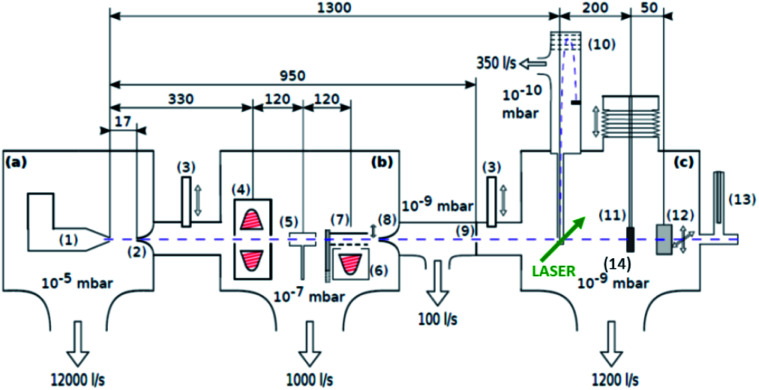
Schematics of the three chamber setup ((a) SC, (b) PC, (c) DC) of the He_*N*_ beam machine used for the experiments described in this paper, see also ref. 22. The droplet beam produced by the cooled nozzle (1) is shaped by the skimmers (2, 8) and an aperture (9). The droplets can be sequentially doped using different sources (4, 5, 6); a beam flag (7) is used to control the pickup (dashed line indicates closed position). For diagnostics, a TOFMS (10), substrates for *ex situ* TEM investigations (11), and a quartz crystal microbalance (12) are available on axis, as well as an off-axis QMS (13). The TOFMS is equipped with an electron gun for electron impact ionization (not shown) but also allows photoionization using an external laser. A UHV-transfer system (14) enables the transport of surface deposited samples to external diagnostics without breaking the vacuum. Reproduced from ref. 22 [P. Thaler, A. Volk, D. Knez, F. Lackner, G. Haberfehlner, J. Steurer, M. Schnedlitz and W. E. Ernst, *J. Chem. Phys.*, 2015, **143**, 134201], with the permission of AIP Publishing.

The experimental setup is depicted in Fig. 1 and described in more detail in ref. 19 and 22. The most important aspects are summarized in the following: highly purified helium is expanded at a stagnation pressure of 20 bar through a 5 μm nozzle, with a temperature of 5.4 K, forming a beam of superfluid He droplets (He_*N*_). The produced droplets have an internal temperature of 0.4 K, which makes them ideal for preserving the structures of dopants and allows to synthesise weakly bound aggregates not achievable otherwise. Due to the He environment chemical interactions can be excluded. The droplet beam is directed through two pick-up zones, where the desired elements are placed in heating crucibles (see sketch in Fig. 1). The crucibles are resistively heated until the vapour pressure of the material is sufficient, such that enough atoms/molecules are picked up by the helium droplets, typically around 10^−3^ mbar. The superfluidity of the He_*N*_ environment enables an unobstructed and frictionless roaming of dopant atoms or molecules. Upon statistical collisions within the He_*N*_, the dopants accumulate and start to form clusters.^23^ For the described experiment, gold (Ögussa 99.9%) was placed in the first pickup oven and V_2_O_5_ (MaTeck 99.9%) powder in the second one. Subsequently, the doped helium beam passes through the UHV chamber with various molecular beam diagnostics like time-of-flight and quadrupole mass spectrometers. It is then terminated on a heatable substrate, suitable for Scanning Transmission Electron Microscopy (STEM) (DENSsolutions Nano-Chip XT carbon) analysis. For the purpose of core–shell cluster creation, we usually take care that the number ratio of core to shell particles is smaller than one to ensure a complete coating of the shell. The dopant numbers cannot be determined quantitatively but we have described a procedure for an estimate based on the shrinkage of the helium droplets after doping.^24^

After deposition, the samples were taken out of the UHV and kept at ambient conditions for several minutes during transport to the microscope. For STEM analysis, a probe-corrected FEI Titan^3^ G2 60-300 was used, set up for High-Angle Annular Dark-Field (HAADF) imaging. The electron energy was set to 300 keV and the convergent angle was 19.6 mrad. The predominant Rutherford scattering in this imaging mode results in a contrast which is highly dependent on the atomic number, causing Au to appear brighter than vanadium oxide. Note that a transition between the oxidation states of vanadium oxides is well described in the literature and is induced by several effects, *e.g.* by electron bombardment and changes in temperature.^25–28^ Therefore, the exposure time for all STEM measurements in this work is reduced to 2.4 μs (the minimum in this mode), if not stated differently. Elemental maps are obtained by EDX and EELS analyses.

## 3 Computational approach

Despite the fact that the details of the growth process of mixed-metallic impurities in helium nanodroplets are still an open debate in the community, there is consent and experimental evidence^19^ that sequential doping is preferably leading to layered structures, which, due to the isotropy of the growth process, translates into *e.g.* core–shell structures for bimetallic setups. Little is known about scenarios involving metals and metal oxides; however, the substantially different character of the chemical bond in terms of directionality, strength and structural parameters makes any predictions difficult. From the theoretical point of view, a first step is the comparison of binding energies per particle for the two components Au and V_2_O_5_. The presence of vanadium(v) oxide is assumed here since it could be confirmed^20^ as the typical oxide forming in helium droplets after pickup of V_2_O_5_ fragments in the helium beam experiment. Note that, in the following computational study, the term ‘unit’ refers to a single Au atom in the case of gold clusters, while it refers to a whole V_2_O_5_ unit in the case of the vanadium(v) oxide clusters.

Our computational approach for the free-gas oligomers is based on the combination of the GFN-xTB method of Grimme,^29^ a less expensive method for pre-optimization and structural search based on a tight binding approach, with the application of density functional theory. For the latter part, the ωB97X-V functional,^30^ a range-separated hybrid GGA density functional with VV10 nonlocal correlation,^31^ as it is implemented in the Q-Chem program package,^32^ is used. For O we use the polarized triple-zeta basis set of Weigend and Ahlrichs,^33^ for V and Au the effective core potential and basis sets of the LANL family.^34^

We define the binding energy per unit as1
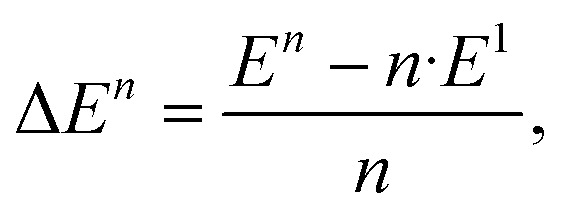
with *E*^*n*^ denoting the total energy of a cluster consisting of *n* units, and calculate this value for clusters of up to six units, *i.e.* for Au_6_ and (V_2_O_5_)_6_. Our results are summarized in Table 1. In this size regime, all Au clusters prefer a flat geometry^35,36^ and the lowest spin multiplicity possible. The corresponding geometries of the vanadium(v) clusters can be found in our previous study on vanadium oxide condensation in helium droplets, see ref. 20 for details.

**Table 1:** Binding energies (in eV) per unit for Au_*n*_ and (V_2_O_5_)_*n*_ with *n* = 2–6, calculated with the ωB97X-V density functional

*n*	2	3	4	5	6
Au_*n*_	−0.95	−0.95	−1.29	−1.43	−1.69
(V_2_O_5_)_*n*_	−3.55	−4.00	−4.22	−4.30	−4.30

**Fig. 2:**
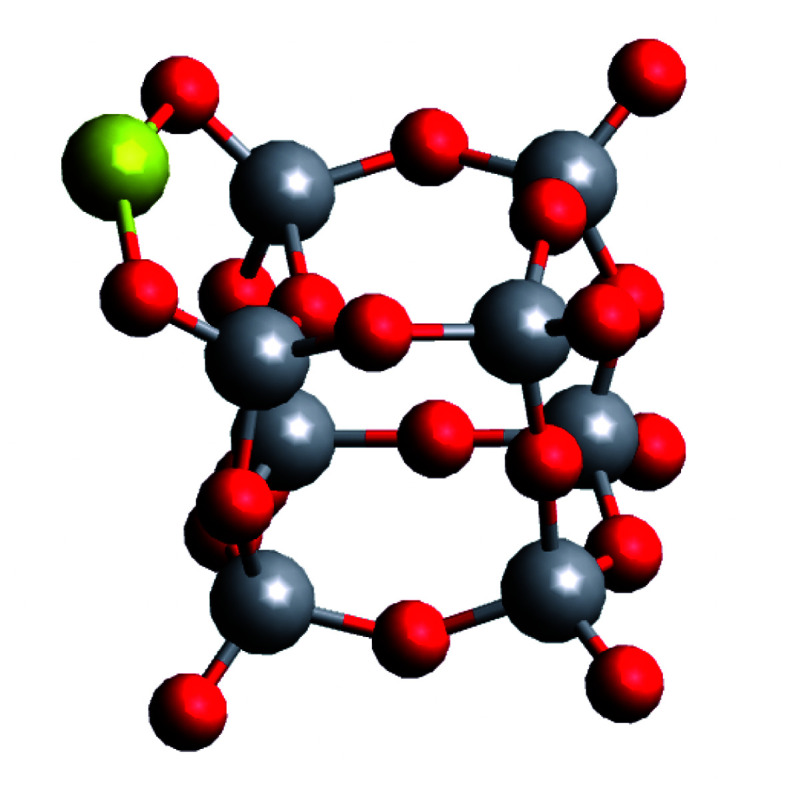
Adsorption of a single Au atom onto a (V_2_O_5_)_4_ cluster, calculated with the ωB97X-V density functional.

Table 1 shows an almost converged energy per unit for vanadium(v) oxide at *n* = 6, while the energy per Au atom is still far from convergence to the bulk value of cohesion energy per particle (3.8 eV experimentally,^37^ 3.1–3.5 eV in theory^35,38,39^) at this size for the metal cluster. It further reveals a substantial difference in binding energies per unit for the two compounds. It is interesting to compare these values to a representative interaction energy between the two components, *e.g.* the binding energy of a single Au atom to (V_2_O_5_)_4_, for which we obtain a value of *E*_i_ = 3.30 eV with the same computational approach. The corresponding geometry of this mixed structure is depicted in Fig. 2. The Au atom prefers a bridging position between two dangling oxygen atoms. This finding of an interaction energy that is in magnitude higher than the bindingenergy per atom of a small Au cluster but smaller than the energy per unit for vanadium(v) oxidecluster formation, suggests that single Au atoms will prefer direct contact with the vanadium oxideover homonuclear aggregation, while free vanadium oxide fragments should prefer to contribute to the formation of larger vanadium oxide structures. On the other hand, this interaction energy is comparable to the binding energy of a single Au atom in bulk, or, in other words, the cohesion energy per atom of solid gold. This suggests a certain competition for the placement of gold atoms near interfaces of the two components: Au adsorption onto V_2_O_5_ units might become energetically more favorable than homonuclear coagulation among gold atoms, if these atoms are only loosely connected to the main gold cluster, for example at edges.

In the process of our mixed cluster formation, this kind of competition should only represent an exception. As observed in all our preceding experiments for the generation of clusters of two materials in helium droplets, the formation of a cluster of thousands of atoms of the material picked up in the first cell has been finished in the millisecond flight time before the second material is added. This means that in the current case, a gold cluster of about 2000 atoms has aggregated when V_2_O_5_ units, presumable in the form of dimers,^20^ are added one by one. In this case, the situation seems to amount to a question about ‘nano-wetting’ of the gold cluster surface by (V_2_O_5_)_2_. Still comparing with our previous experience about core–shell formation, we would expect a similar result for the newly formed Au–V_2_O_5_ particles. While we cannot give precise numbers of our dopant particles, our experimental procedure always aims at a relation of the number of shell particles to core particles larger than one to ensure a full coating. From the theoretical side, calculations of binding energies of V_2_O_5_ molecules and/or dimers to gold cluster surfaces or at least to Au(111) would be desirable but are not available to our knowledge. Observations by the Freund group^40^ during the experimental creation of thin V_2_O_5_ films on Au(111) indicated island formation due to coalescence at preferred locations. Although their experiments differ from our procedures, there remains the question whether our coating by subsequent doping will work as in the case of two metals or result in nano-islands.

The above calculations for individual units will become relevant when the dynamics after cluster deposition and the thermal and electron beam treatment have started. Then situations may occur where diffusion or migration of individual gold atoms leads to the described bond competition.

## 4 Experimental results and discussion

**Fig. 3:**
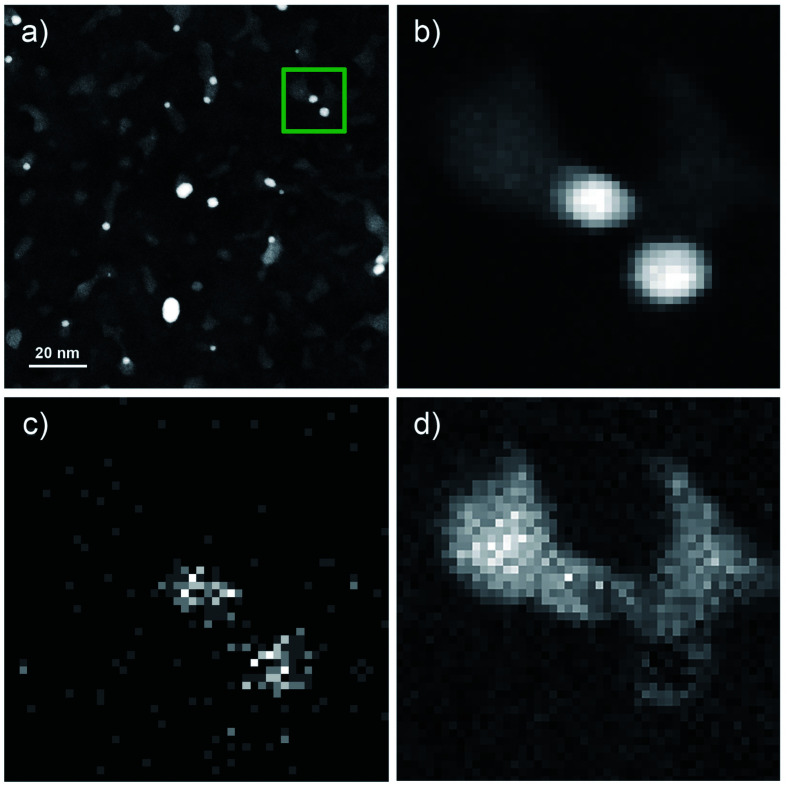
Overview of STEM HAADF images of V_2_O_5_/Au structures. (b) HAADF region for the measured EELS and EDX spectroscopy. (c) Spatially resolved EDX analysis based on the Au M_α_ peak at 2.1 keV. (d) Spatially resolved elemental EELS analysis using the signal of the V L-edge. (b), (c), and (d) are magnified images of the green marked area in (a), a square 20 nm x 20 nm.

Fig. 3 shows the results of the analytical STEM investigations of the particles. The high angle annular dark field images in Fig. 3a and b provide a contrast which depends on the atomic number. Therefore, bright regions exhibit very small Au clusters with sizes between 2 and 5 nm, the medium gray regions are the vanadium oxide deposits. In addition, an EELS and EDX-spectrum image has been recorded in parallel^41^ on the structure surrounded by the green frame in Fig. 3a. The magnified HAADF image of this area is given in Fig. 3b. The EDX elemental map of gold is displayed in Fig. 3c using the signal of the Au M line at 2.2 keV, thus confirming that the bright particles are indeed Au clusters. In Fig. 3d we have the EELS elemental map showing the vanadium distribution recorded with the V-L_2,3_ ionization edge which correlates with the medium gray regions in the HAADF image of Fig. 3b thus highlighting the vanadium oxide phase. Due to the overlapping of the V-L_2,3_ edge with the O K edge it was not possible to record oxygen maps using the O K edge.^42^ Although the near edge-fine structures (ELNES) of the EELS signal has been successfully employed for the identification of vanadium oxides and their oxidation states in previous experiments on vanadium oxide particles as well as thin films,^43^ we could not employ this method for the identification of the vanadium oxidation state in our STEM measurements. In order to avoid particle damage and to prevent electron beam induced reduction, we have to use a low dose electron beam and extremely small exposure times for all STEM measurements in this work. We reduced the exposure time to 2.4 μs which is the minimum in this mode. Therefore, we have a rather weak EELS signal and a lower spatial and energy resolution compared to other published spectra.^42,43^

Nonetheless, our previous measurements of vanadium oxide nanoparticles alone under the same conditions and on the same substrates, were shown to be pure V_2_O_5_ nanostructures,^21^ therefore we identify these clusters as Au–V_2_O_5_. When inspecting the overview image in Fig. 3a, the Au share of each structure does not seem to be encapsulated by the larger V_2_O_5_ part of any structure. Our previous two-component metal cluster aggregation had yielded core@shell structures such as Au@Ag, Co@Au, Ni@Au, Fe@Au, and Ag@ZnO.^44–49^ Therefore, we expected an Au core surrounded by a V_2_O_5_ shell, but in the current images, most Au clusters are connected to larger vanadium structures at their tail, while a complete coating is never observed.

**Fig. 4:**
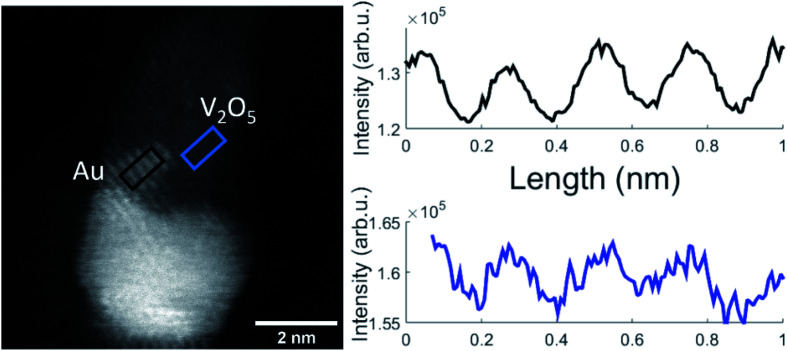
STEM HAADF images of a V_2_O_5_/Au structure (left) and the resulting lattice distances of the nanostructure extracted from contrast scans (right). Here the V_2_O_5_ part of the Janus-type particle (blue rectangle) exhibits a lattice spacing corresponding to a V_2_O_5_(211) orientation (2.48 Å literature value) which is close to the Au(111) lattice plane separation of 2.36 Å as it is retrieved from the black rectangle.

A representative Au–V_2_O_5_ cluster is depicted in Fig. 4. The bright lower part of the structure can be assigned as Au and the darker part on top as V_2_O_5_ portion. Within both structures the lattice plane distances are visible as periodic lines. Only planes that are oriented parallel to the incoming electron beam can be clearly identified. Some portions of the image appear washed-out and do not allow lattice structure assignments due to tilted angles with respect to the beam direction. Intensity plots for the areas in the black rectangle, assigned as thin Au layer, and the blue marked area, assigned as vanadium oxide, are shown in the right-hand part of Fig. 4. Literature values for cubic Au(111) and orthorhombic V_2_O_5_(211) are 2.36 Å and 2.48 Å, respectively, which lets us choose these assignments in reasonable agreement within the spatial resolution of 0.1 Å of the microscope. In our work on pure vanadium oxide depositions from the helium droplet source,^21^ corresponding STEM images of V_2_O_5_ clusters exhibited (020), (011), and (200) lattice portions with 2.2 Å, 2.7 Å, and 5.7 Å lattice distances, respectively. We have no control of the nanocrystal orientation. Furthermore, the contrast of the vanadium oxide part is not high enough for 3D imaging, a technique which we had demonstrated for Au@Ag core@shell clusters with our instrument before.^50^ As it also turns out, the Au–V_2_O_5_ particles alter their shape during longer electron beam exposure as previously shown for other transition metal oxide particles which have been also prepared by the helium droplet method.^51^ This makes a reconstruction of a 3D image from sequentially taken exposures unreliable. In the past, V_2_O_5_ films have been grown on plane Au(111) surfaces by oxidation of a previously deposited V layer with a sufficiently high pressure of O_2_.^28^ In our experiments, primarily V_2_O_5_ dimers are added one by one to a gold cluster core grown inside the droplets before,^20^ without the need for subsequent oxidation. As shown in a model calculation,^52^ the kinetic energy of the impact of the droplets on the substrate surface is small compared to the binding energy between the atoms and molecules of the grown cluster. Therefore, we believe that the observed V_2_O_5_ portions represent regular nanocrystals with typical literature lattice constants.

**Fig. 5:**
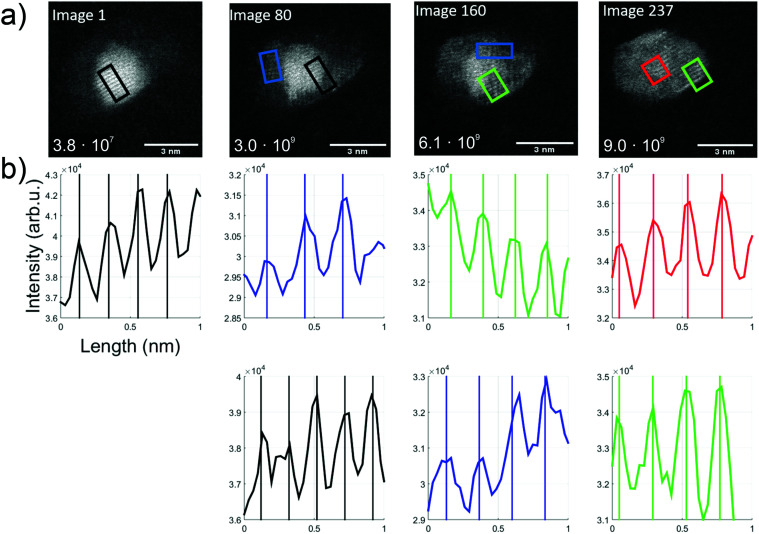
Overview over the thermal kinetics in an Au/V_2_O_3_ cluster at constant 650 °C. (a) A time series of TEM images (video provided in the ESI†). The number in the left lower corner indicates the estimated dosage in units of total electrons per cluster area. (b) Intensity profiles, extracted from the images directly above, and printed in matching colors to indicate the corresponding rectangular areas in the TEM images (length about 1 nm). Periodicities are determined *via* fits.

For further understanding of this Au/V_2_O_5_ nanosystem, *in situ* heating was performed with a MEMS-based heating chip during the STEM measurement.^53^ A Janus-type particle of gold and vanadium oxide was monitored upon heating and appeared to maintain the described Au and V_2_O_5_ lattice parts stably up to 300 °C. Changes were observed when the samples were further heated *in situ* up to a temperature of 650 °C during the STEM measurements. In Fig. 5 several subsequently taken images of a structure at 650 °C are shown. Note that for each image within this figure the temperature was kept constant at 650 °C, and every image represents approximately one second of collection time. Therefore, 80 pictures means that approximately 80 seconds have passed after the first image was produced at this high temperature. For the calculation of the total electron illumination dose of the nanoparticle area, the algorithm described in ref. 51 was used. According to this estimate, the cluster had already been exposed to 3.8 × 10^7^ electrons when the first image was taken, and for every 80 seconds of additional exposure the same number of electrons per second can be assumed if the projected area is approximately constant. In the 237 s from the beginning to the end of this sequence, a significant change is observed and a hexagonal shape seems to evolve with a partial coating of the gold portion with vanadium oxide.

The first image shows the typical Au fcc lattice in (200) orientation with a plane distance of 2.1 Å, while the second image shows V_2_O_3_(104) lattice planes with 2.7 Å (blue) and Au(200) planes with 2.1 Å. In the third image, probed by two rectangular selections (green and blue), a lattice distance of 2.3 Å can be identified which indicates the V_2_O_3_(006) plane. In the last image, the formation of 120° angles seems to start, and a spacing of 2.4 Å is measured within the red and green rectangles, which could refer to the Au(111) and/or V_2_O_3_(111) plane.

With the lattice plane separations of V_2_O_5_ and V_2_O_3_ being such that one can find orientations for either one of them with the determined values, an unambiguous assignment is difficult. For example, 2.7 Å distance can be assigned to V_2_O_3_(104) but also to V_2_O_5_(011). We assume that our vanadium oxide samples show the same thermal reduction behaviour as observed by other groups who report a reduction from V_2_O_5_ to V_2_O_3_ in TEM studies for temperatures above 600 °C. Su and Schlögl have suggested a transition from V_2_O_5_*via* VO_2_ to V_2_O_3_, where V_2_O_3_ remained at 600 °C (ref. 25) and Ramana *et al.* did not observe any reduced phase below 450 °C. At 500 °C they determined a phase which contained mainly V_2_O_5_ and additional, smaller amounts of V_2_O_3_ and V_4_O_9_.^27^ After heating beyond 600 °C only V_2_O_3_ remained. Both groups reported an ongoing reduction, predicting that V_2_O_3_ prevails at temperatures above 600 °C. For this reason, we assign the measured lattice plane separations in the bright areas to Au and the others to V_2_O_3_. The image sequence indicates dynamics that may be induced by the electron beam exposure, from image 1 with already more enclosure of the gold part by vanadium oxide (in comparison to the room temperature picture in Fig. 3), to a change of the gold cluster shape with some hexagonal boundaries and a more pronounced vanadium oxide coating.

**Fig. 6:**
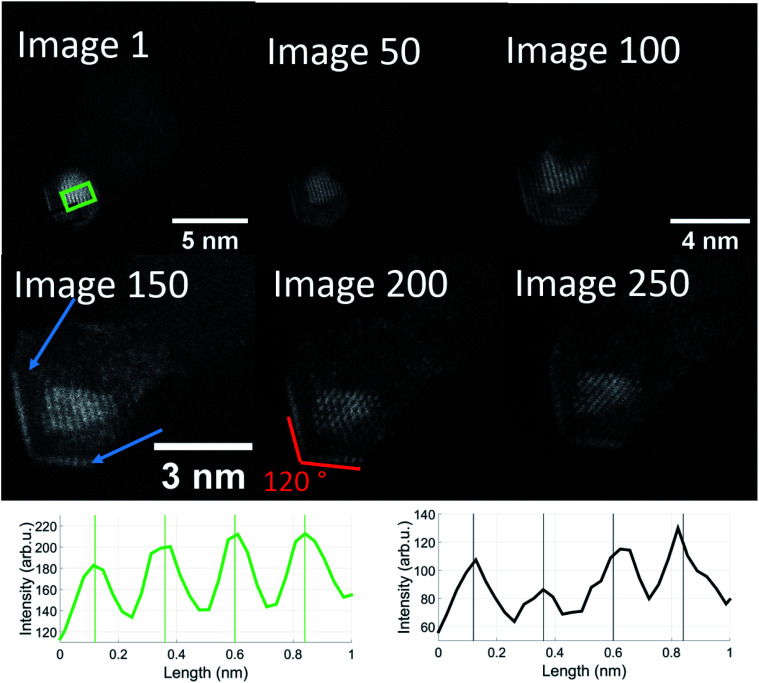
Sequential images taken from the same Au/V_2_O_3_ nanostructure at a temperature of 650 °C. Two distinct features can be observed: the Au atoms form a monolayer on the surface of the structure, marked by blue arrows and additionally, these layers as well as the gold core form an angle of exactly 120 degrees. The images were taken from a video which is included in the ESI.† The magnification was increased after image 50 and again after image 100. Note that the enhanced diffusion of the Au atoms has already started in image 1. The onset of a monolayer can be observed in the left corner. The lattice plane distances in the green and black rectangles are close to 2.4 Å, similar to image 237 of Fig. 5, with the difference that the gold and vanadium oxide parts seem more strongly separated, *i.e.* they presumably correspond to Au(111) (green) and V_2_O_3_(111) (black).

In order to follow the described dynamics further, we produced a movie sequence on a different cluster which we started after a visual state similar to image 237, maintaining the same electron dose at constant 650 °C for another 250 seconds. Fig. 6 shows a number of images from this movie where the length scale is shown in image 1. Beyond image 50 a magnification is applied in order to make the structure changes more visible. Already in image 1, the structural feature of 120 degree angles becomes more emphasized. 50 s later (image 50), the brighter 120 degree edges indicate some gold diffusion which then increases to cover the edges such that we see a primary gold core with the typical Au(111) lattice plane separation of 2.36 Å, a vanadium oxide part surrounding the gold, perhaps enclosing it entirely, and a gold monolayer outside due to diffusion. The image sequence indicates a significant impact of our electron dose of roughly 10^7^ electrons per second on the diffusion processes towards a partial phase segregation of the gold and vanadium oxide parts. The 120° edges suggest a particular change of the gold cluster shape. According to ref. 54, metal nanoparticles with diameters in the range of 15 nm or below, favour a so-called Wulff shape for energy reasons. A gold cluster would then arrange as a truncated octahedron with 6 square and 8 hexagon surfaces and with 36 edges and 24 vertices (point group *O*_h_, sphericity 0.9099). While such interpretation is somewhat speculative, it may provide an explanation for the structural change upon the impact of thermal and electron beam exposure. In our previous temperature studies of metal core@shell nanoparticles including our model calculations, we have seen different diffusion processes. In the case of Au@Ag and Ag@Au,^44^ we observe nanoalloys at high temperature; in Ni@Au, nickel diffuses through the gold protective cover,^45^ as does Fe in Fe@Au.^46^ Among the iron triad combined with a gold shell, increased temperature tends to promote gold diffusion in Co@Au such that the formerly spherical Au shell becomes thinner on one side of the Co core and thicker on the other.^48,55^ So far, this is just a qualitative comparison. The binding energies of a gold atom to a gold cluster and a (V_2_O_5_)_2_ unit to a V_2_O_5_ cluster^20^ are comparable with about 3.5 to 4 eV, but the Au–VO system is much more complex and the reduction from V_2_O_5_ to V_2_O_3_ – perhaps even through intermediate oxidation states – will influence the diffusion dynamics significantly.

## 5 Summary

Gold–vanadium oxide clusters of about 10 nm diameter were grown inside superfluid helium nanodroplets in a molecular beam expansion and deposited on amorphous carbon substrates. The samples were subsequently studied by scanning transmission electron microscopy, electron energy loss spectroscopy, and energy-dispersive X-ray spectroscopy over a temperature range from room temperature to 650 °C. At 650 °C, the diffusion dynamics inside individual clusters on the surface was monitored *via* STEM imaging. Several interesting features and processes were observed. In contrast to our metal core@shell helium droplet assisted cluster production, the Au–VO nanostructures exhibit rather a Janus type particle character. Thanks to our high resolution instrument, lattice planes could be identified for the nanocrystals and assigned. At room temperature, V_2_O_5_ cluster structures were assigned similar to our earlier investigations.^21^ Above 600 °C, a reduction to V_2_O_3_ was assumed due to other studies on VO films,^26–28,43^ and corroborated by our lattice plane distance measurements on the deposited structures.

Continued electron beam exposure at 650 °C led to a restructuring of the whole nanoparticle towards a hexagonal shape, which we interpret as the formation of an Au Wulff-shaped cluster with a surrounding vanadium oxide layer and an Au-rich surface layer.

## Conflicts of interest

There are no conflicts to declare.

## Supplementary Material

FD-242-D2FD00089J-s001

## References

[cit1] Roduner E. (2006). Chem. Soc. Rev..

[cit2] Jimenez-Izal E., Gates B. C., Alexandrova A. N. (2019). Phys. Today.

[cit3] Zhang Q., Lee I., Joo J. B., Zaera F., Yin Y. (2013). Acc. Chem. Res..

[cit4] Astruc D. (2020). Chem. Rev..

[cit5] Li X.-N., Wang L.-N., Mou L.-H., He S.-G. (2019). J. Phys. Chem. A.

[cit6] Hu M., Chen J., Li Z.-Y., Au L., Hartland G. V., Li X., Marquez M., Xia Y. (2006). Chem. Soc. Rev..

[cit7] Rosi N. L., Giljohann D. A., Thaxton C. S., Lytton-Jean A. K. R., Han M. S., Mirkin C. A. (2006). Science.

[cit8] Wan F., Niu Z. (2019). Angew. Chem., Int. Ed..

[cit9] Rogov A., Mugnier Y., Bonacina L. (2015). J. Opt..

[cit10] Osada M., Sasaki T. (2009). J. Mater. Chem..

[cit11] Liu M., Su B., Tang Y., Jiang X., Yu A. (2017). Adv. Energy Mater..

[cit12] Baddour-Hadjean R., Safrany Renard M., Emery N., Huynh L., Le M., Pereira-Ramos J. (2018). Electrochim. Acta.

[cit13] Baddour-Hadjean R., Safrany Renard M., Pereira-Ramos J. (2019). Acta Mater..

[cit14] Andreeva D., Nedyalkova R., Ilieva L., Abrashev M. (2004). Appl. Catal., B.

[cit15] Camposeco R., Zanella R. (2022). Catal. Today.

[cit16] Yang X., Wang W., Wang C., Xie H., Fu H., An X., Jiang X., Yu A. (2018). Powder Technol..

[cit17] Schmid G., Chi L. F. (1998). Adv. Mater..

[cit18] Palmer R. E., Cai R., Vernieres J. (2018). Acc. Chem. Res..

[cit19] Ernst W. E., Hauser A. W. (2021). Phys. Chem. Chem. Phys..

[cit20] Lasserus M., Schnedlitz M., Messner R., Lackner F., Ernst W. E., Hauser A. W. (2019). Chem. Sci..

[cit21] Lasserus M., Knez D., Lackner F., Schnedlitz M., Messner R., Schennach D., Kothleitner G., Hofer F., Hauser A. W., Ernst W. E. (2019). Phys. Chem. Chem. Phys..

[cit22] Thaler P., Volk A., Knez D., Lackner F., Haberfehlner G., Steurer J., Schnedlitz M., Ernst W. E. (2015). J. Chem. Phys..

[cit23] Hauser A. W., Volk A., Thaler P., Ernst W. E. (2015). Phys. Chem. Chem. Phys..

[cit24] Volk A., Thaler P., Knez D., Hauser A. W., Steurer J., Grogger W., Hofer F., Ernst W. E. (2016). Phys. Chem. Chem. Phys..

[cit25] Su D. S., Wieske M., Beckmann E., Blume A., Mestl G., Schlögl R. (2001). Catal. Lett..

[cit26] Su D. S., Schlögl R. (2002). Catal. Lett..

[cit27] Ramana C., Utsunomiya S., Ewing R., Becker U. (2006). Solid State Commun..

[cit28] Guimond S., Göbke D., Romanyshyn Y., Sturm J. M., Naschitzki M., Kuhlenbeck H., Freund H.-J. (2008). J. Phys. Chem. C.

[cit29] Grimme S., Bannwarth C., Shushkov P. (2017). J. Chem. Theory Comput..

[cit30] Mardirossian N., Head-Gordon M. (2014). Phys. Chem. Chem. Phys..

[cit31] Mardirossian N., Head-Gordon M. (2014). J. Chem. Phys..

[cit32] Shao Y. (2015). et al.. Mol. Phys..

[cit33] Weigend F., Ahlrichs R. (2005). Phys. Chem. Chem. Phys..

[cit34] Roy L. E., Hay P. J., Martin R. L. (2008). J. Chem. Theory Comput..

[cit35] Assadollahzadeh B., Schwerdtfeger P. (2009). J. Chem. Phys..

[cit36] Rincon L., Hasmy A., Marquez M., Gonzalez C. (2011). Chem. Phys. Lett..

[cit37] Kittel C. , Introduction to Solid State Physics, Wiley, 8th edn, 2004

[cit38] Takeuchi N., Chan C. T., Ho K. M. (1989). Phys. Rev. B: Condens. Matter Mater. Phys..

[cit39] Heimel G., Romaner L., Brédas J.-L., Zojer E. (2006). Surf. Sci..

[cit40] Guimond S., Sturm J. M., Göbke D., Romanyshyn Y., Naschitzki M., Kuhlenbeck H., Freund H.-J. (2008). J. Phys. Chem. C.

[cit41] Kothleitner G., Neish M. J., Lugg N. R., Findlay S. D., Grogger W., Hofer F., Allen L. J. (2014). Phys. Rev. Lett..

[cit42] Su D., Zandbergen H., Tiemeijer P., Kothleitner G., Hävecker M., Hébert C., Knop-Gericke A., Freitag B., Hofer F., Schlögl R. (2003). Micron.

[cit43] Hébert C., Willinger M., Su D. S., Pongratz P., Schattschneider P., Schlögl R. (2002). Eur. Phys. J. B.

[cit44] Lasserus M., Schnedlitz M., Knez D., Messner R., Schiffmann A., Lackner F., Hauser A. W., Hofer F., Ernst W. E. (2018). Nanoscale.

[cit45] Schnedlitz M., Lasserus M., Meyer R., Knez D., Hofer F., Ernst W. E., Hauser A. W. (2018). Chem. Mater..

[cit46] Lasserus M., Knez D., Schnedlitz M., Hauser A. W., Hofer F., Ernst W. E. (2019). Nanoscale Adv..

[cit47] Schnedlitz M., Fernandez-Perea R., Knez D., Lasserus M., Schiffmann A., Hofer F., Hauser A. W., de Lara-Castells M. P., Ernst W. E. (2019). J. Phys. Chem. C.

[cit48] Schnedlitz M., Knez D., Lasserus M., Hofer F., Fernández-Perea R., Hauser A. W., de Lara-Castells M. P., Ernst W. E. (2020). J. Phys. Chem. C.

[cit49] Schiffmann A., Jauk T., Knez D., Fitzek H., Hofer F., Lackner F., Ernst W. E. (2020). Nano Res..

[cit50] Haberfehlner G., Thaler P., Knez D., Volk A., Hofer F., Ernst W. E., Kothleitner G. (2015). Nat. Commun..

[cit51] Knez D., Schnedlitz M., Lasserus M., Schiffmann A., Ernst W. E., Hofer F. (2018). Ultramicroscopy.

[cit52] Thaler P., Volk A., Ratschek M., Koch M., Ernst W. E. (2014). J. Chem. Phys..

[cit53] Krisper R., Lammer J., Pivak Y., Fisslthaler E., Grogger W. (2022). Ultramicroscopy.

[cit54] Barmparis G. D., Lodziana Z., Lopez N., Remediakis I. N. (2015). Beilstein J. Nanotechnol..

[cit55] Knez D., Schnedlitz M., Lasserus M., Hauser A. W., Ernst W. E., Hofer F., Kothleitner G. (2019). Appl. Phys. Lett..

